# Complete mitochondrial genome sequence of the high-altitude Brazilian tree frog *Bokermannohyla alvarengai* (Anura, Hylidae)

**DOI:** 10.1080/23802359.2017.1325339

**Published:** 2017-05-13

**Authors:** Nathália Gonçalves da Silva Lima, Anderson Oliveira do Carmo, Ana Paula Vimieiro Martins, Rafael Cerqueira Castro de Souza, Evanguedes Kalapothakis, Paula Cabral Eterovick

**Affiliations:** a Departamento de Biologia Geral, Instituto de Ciências Biológicas, Universidade Federal de Minas Gerais, Belo Horizonte, Minas Gerais, Brasil;; b Programa de Pós Graduação em Biologia de Vertebrados, Pontifícia Universidade Católica de Minas Gerais, Belo Horizonte, Minas Gerais, Brasil

**Keywords:** Mitochondrion, mtDNA, next-generation sequencing, Neobatrachia, treefrog

## Abstract

The first complete mitochondrial genome (mtDNA) for the genus *Bokermannohyla* (Hylidae) is presented. The mtDNA contains 17,325 bp and is similar in size, gene content, and gene location to other hylid mitochondrial genomes described, with 2 rRNA, 22 tRNA, and 13 PCGs. The control region (D-loop) is shorter than in mtDNAs of hylids from Asia. A phylogenetic tree based on homologous genes did not corroborate the monophyly of Hylidae neither the recently proposed monophyly of *Hyla* and *Dryophytes*.

*Bokermannohyla alvarengai* (Hylidae) is a stream tree frog restricted to the highlands of the Espinhaço Mountain Range in southeastern Brazil, a region with high amphibian diversity, endemism, past events of vicariance, and isolated speciation (Leite et al. 2008; Ramos 2017). These tree frogs have unusual adaptations that include skin lipids secreted to help regulate heat exchange and hydric balance (Tattersall et al. 2006; Centeno et al. 2015).

At GenBank, only six complete mtDNAs are available for the family Hylidae, all from Asian species. Other two incomplete mtDNAs are available for species from South America (*Callimedusa tomopterna* (JX564887) and *Osteocephalus taurinus* (JX564881)). Here, we describe the first mtDNA for the genus *Bokermannohyla*.

Total genomic DNA was extracted (Herrmann & Frischauf 1987) from muscle tissue of one specimen of *B. alvarengai* collected at Serra do Cipó, at the southern portion of Espinhaço (19°12′-19°20'S, 43°30′-43°40′W). Remaining tissue is at UFMG tissue collection (UFMG BDT AN1700000). Permits were provided by IBAMA/SISBIO (48825-2).

A genomic library was prepared using Nextera DNA Sample Preparation Kit (Illumina, San Diego, CA) and sequenced in a MiSeq (Illumina) sequencer using MiSeq Reagent Kit v3 600 and paired-end strategy. Primer walking and amplicon cloning with Topo vector in *Escherichia coli* XL1-Blue (Phoneutria) were also employed to fill a gap of 20 bp, with sequencing in ABI 3130 (Applied Biosystems, Foster City, CA). *De novo* assembly was conducted in CLC Genomics Workbench 9.0 (Bio-Qiagen, Aarhus, Denmark) and final mtDNA sequence was annotated using MITOS (Bernt et al. 2013). The complete annotated *B. alvarengai* mtDNA was compared with complete mtDNAs from 13 anuran species available at GenBank using Mega 6 (Tamura et al. 2013), with the neighbour-joining algorithm (Figure 1).

**Figure 1.**
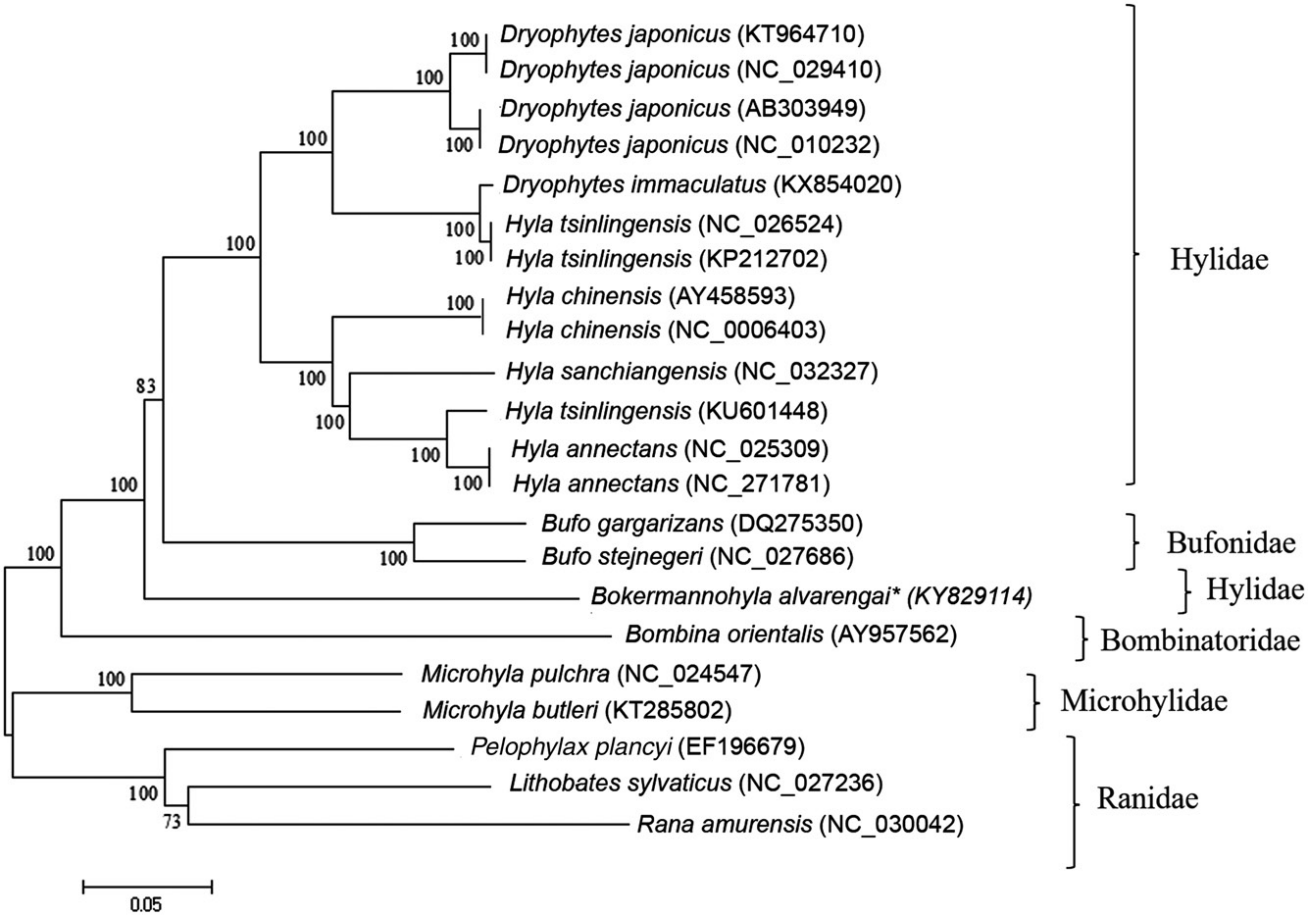
Phylogenetic tree generated using neighbor-joining algorithm based on complete mitochondrial genomes of the anuran species *Dryophytes japonicus*, *D. immaculatus*, *Hyla tsinlingensis, H. chinensis, H. sanchiangensis*, *H. annectans*, *Bokermannohyla alvarengai*, *Bufo gargarizans*, *B. stejnegeri*, *Bombina orientalis*, *Microhyla pulchra*, *M. butleri*, *Pelophylax plancyi*, *Lithobates sylvaticus,* and *Rana amurensis*. The phylogenetic tree was constructed under the Kimura-2 parameter model and consensus tree using 1000 bootstrap. Numbers indicate support of each clade.

The mtDNA of *B. alvarengai* (GenBank accession number KY829114) is similar to other hylids’ concerning size, gene content, and gene distribution. It is a circular molecule of 17,325 bp with 41.1% GC, 37 coding sequences, and a non-coding region. Except for eight tRNAs and ND6, sequences are coded in the heavy strand. Start codon ATG was found in 12 PCGs. Only the COI gene has ATA as the start codon, similar to *Dryophytes japonicus* (Qinglin et al. 2017)*, Hyla annectans* (Ye et al. 2016), *H. tsinlingensis* (Huang et al. 2016), *H. sanchiangensis*, and *H. chinensis* (Chen et al. 2016). COII, ND3, and ND6 have AGA as stop codon. ND1, ND2, COI, and Cytb present TAG, and ND5 has AGG as the stop codon. The remaining genes have TAA as the stop codon.

The control region (D-loop) is smaller than the ones of *D. japonicus* and *D. immaculatus*. The first 600 bp of the D-loop of *B. alvarengai* were more than 74% similar to other anuran species. The phylogenetic tree comparing the available complete anuran mtDNA sequences disagrees with the monophyly of Hylidae (Frost et al. 2006) as *Bufo* species are inserted between *B. alvarengai* and the other hylids. It also does not support the monophyly of the genera *Hyla* as posteriorly separated from *Dryophytes* by Duellman et al. (2016).
